# Clinical and pharmacokinetic evaluation of S-ketamine for intravenous general anaesthesia in horses undergoing field castration

**DOI:** 10.1186/s13028-015-0112-4

**Published:** 2015-05-03

**Authors:** Daniela Casoni, Claudia Spadavecchia, Beat Wampfler, Wolfgang Thormann, Olivier L Levionnois

**Affiliations:** Anesthesiology and Pain Therapy Division, Department of Veterinary Clinical Science of the Vetsuisse Faculty of Berne, Länggassstrasse 124, CH-3012 Berne, Switzerland; Nationales Pferdezentrum Bern, Mingerstrasse 3, CH-3000 Berne, Switzerland; Clinical Pharmacology Laboratory, Institute for Infectious Diseases, University of Berne, Murtenstrasse 35, CH-3010 Bern, Switzerland

**Keywords:** S-ketamine, Horses, Intravenous anesthesia, Field castration, Plasma level, Pharmacokinetic

## Abstract

**Background:**

Intravenous anaesthetic drugs are the primary means for producing general anaesthesia in equine practice. The ideal drug for intravenous anaesthesia has high reliability and pharmacokinetic properties indicating short elimination and lack of accumulation when administered for prolonged periods. Induction of general anaesthesia with racemic ketamine preceded by profound sedation has already an established place in the equine field anaesthesia. Due to potential advantages over racemic ketamine, S-ketamine has been employed in horses to induce general anaesthesia, but its optimal dose remains under investigation. The objective of this study was to evaluate whether 2.5 mg/kg S-ketamine could be used as a single intravenous bolus to provide short-term surgical anaesthesia in colts undergoing surgical castration, and to report its pharmacokinetic profile.

**Results:**

After premedication with romifidine and L-methadone, the combination of S-ketamine and diazepam allowed reaching surgical anaesthesia in the 28 colts. Induction of anaesthesia as well as recovery were good to excellent in the majority (n = 22 and 24, respectively) of the colts. Seven horses required additional administration of S-ketamine to prolong the duration of surgical anaesthesia. Redosing did not compromise recovery quality. Plasma concentration of S-ketamine decreased rapidly after administration, following a two-compartmental model, leading to the hypothesis of a consistent unchanged elimination of the parent compound into the urine beside its conversion to S-norketamine. The observed plasma concentrations of S-ketamine at the time of first movement were various and did not support the definition of a clear cut-off value to predict the termination of the drug effect.

**Conclusions:**

The administration of 2.5 mg/kg IV S-ketamine after adequate premedication provided good quality of induction and recovery and a duration of action similar to what has been reported for racemic ketamine at the dose of 2.2 mg/kg. Until further investigations will be provided, close monitoring to adapt drug delivery is mandatory, particularly once the first 10 minutes after injection are elapsed. Taking into account rapid elimination of S-ketamine, significant inter-individual variability and rapid loss of effect over a narrow range of concentrations a sudden return of consciousness has to be foreseen.

## Background

The administration of racemic ketamine and diazepam following the premedication with an α-2 agonist for producing short term anaesthesia represents one of the most significant events in equine anaesthesia in the last fifty years [1]. Since its introduction in the early Seventies, racemic ketamine has been gaining hedging popularity due to its safety and reliability in allowing most surgical procedures and rapid recoveries. However, undesirable psychomimetic effects during the emergence from general anaesthesia have been reported.

Racemic ketamine is the mixture of two enantiomers: S (+) ketamine and R (−) ketamine: recently, it has been recognized that each ketamine enantiomer can evoke different effects. In humans, when compared to R- and racemic ketamine at clinical anaesthetic doses, S-ketamine was found to evoke shorter and fewer psychomimetic effects after general anaesthesia [2] and for comparable anaesthetic depth the S-enantiomer was associated with a more rapid recovery of psychomotor skills than the racemic mixture [3]. Furthermore, a different pharmacokinetic profile for the two enantiomers was described in humans [4] as well as in dogs and ponies [5,6]. The extent of pharmacokinetic enantioselectivity has been shown to be species-specific and dependent on drugs concomitantly administered [7]. If administered alone, S-ketamine has a higher clearance than in the racemic mixture resulting in quicker elimination, shorter duration of action and faster recovery from anaesthesia [4,8,9].

In horses, ataxia, excitation, and residual muscle rigidity increase the risk for complications during the recovery phase. Since trained assistance is minimal under field conditions, a single injection of an anaesthetic should ideally allow for completion of a standard surgical procedure. To extend the duration of anaesthesia, re-dosing should be possible, but without compromising its safety and overall quality. Due to its favorable pharmacological profile, compared to the racemic ketamine, S-ketamine might result in a smoother quality of induction and in a better quality of recovery. Data in the literature are still insufficient to determining an appropriate anaesthetic protocol based on S-ketamine for surgical anaesthesia in horses. Several studies have shown that the ratio of anaesthetic potency between S-ketamine and racemic ketamine can be up to 1:3 [10-12]. In dogs, a potency ratio of 1:1.3 has been reported under experimental conditions [13] and 1:1.5 under clinical conditions [14]. The results from Rossetti *et al*. [15] suggested a similar potency ratio in horses. In a previous clinical trial 2 mg/kg S-ketamine was found by the authors as being insufficient to obtain surgical anaesthesia (unpublished data).

This study aimed at evaluating whether S-ketamine could be used as a single bolus to provide short-term surgical anaesthesia in horses undergoing castration, and at reporting the pharmacokinetic profile of S-ketamine and its main active metabolite, S-norketamine. We hypothesized that S-ketamine at 2.5 mg/kg given as a single bolus would be appropriate to perform surgical castration in healthy colts under field conditions.

## Methods

This prospective clinical trial was reviewed and approved by the Committee for Animal Experimentation of the Canton Bern, Switzerland (BE 11/43).

### Study design and animals

Twenty-eight colts of various breeds scheduled for field castration, and classified as ASA I or II, older than 6 months and weighing more than 200 kg were included in the study. Weight was calculated in kg via the following formula: (girth^2^× length)/ 11877 [16]. All the animals were fasted for 6 hours before anaesthesia, but free access to the water was granted.

### Experimental protocol

After clinical examination and weight assessment, every horse was premedicated with romifidine at 0.1 mg/kg (Sedivet® Boehringer Ingelheim, Switzerland) injected in the right jugular vein. Five to ten minutes after premedication, a 13 G. indwelling catheter (Provet AG/Petco AG Heiland, Switzerland) was placed in the left jugular vein. Immediately after catheter placement, L-methadone/fenpipramide 50/2.5 μg kg^−1^ IV (Polamivet ®, Intervet, Switzerland) was administered and the horses were walked from their box to the induction area. Sedation quality was scored including response to tactile and auditory stimuli (Table 1) at least 5 minutes after catheter placement and not more than 30 minutes after romifidine administration. If the sedation score was higher than 2, additional romifidine was administered (0.03 mg/kg IV) and the sedation reassessed after 5 minutes. If the sedation score remained higher than 2, a third dose (0.015 mg/kg) was allowed, but if still judged insufficient, the horse would be excluded from the trial. The overall quality of sedation achieved was evaluated using a visual analogue scale (VAS) from 0 (best possible sedation) to 100 mm (worst possible sedation). Heart rate (HR), pulse rate (PR) and respiratory rate (RR) were then recorded.

**Table 1:** **Evaluation of sedation and responsiveness to external stimuli**

**Parameter**	**Score**	**Description**
Sedation score	1 (Profound)	Animal stupourous, CTG < 60 cm., severe ataxia
	2 (Strong)	Animal apathetic, 60 < CTG <80 cm moderate ataxia
	3 (Moderate)	Animal calm, head lowered between 80 < CTG <100 cm, mild ataxia
	4 (Mild)	Animal calm, CGT > 100 cm., minimal ataxia
	5 (Absent)	No apparent sedation
Response to tactile stimulation (Picking the neck)	1 (Absent)	No response
	2 (Mild)	Slow, delayed response
	3 (Moderate)	Moderate response
	4 (Strong)	Rapid, marked response
Response to auditory stimulation (Clapping hands)	1 (Absent)	No response
	2 (Mild)	Slow, delayed response
	3 (Moderate)	Moderate response
	4 (Strong)	Rapid, marked response

A sedation score of 1 or 2 was mandatory before proceeding to induction of general anaesthesia. CGT = chin-to-ground distance (measured in cm).

General anaesthesia was induced with diazepam 0.05 mg/kg IV (Valium, Roche, Basel, Switzerland) and S- ketamine 2.5 mg/kg IV (Keta-S Graeub AG, Bern, Switzerland) administered in sequence. Anaesthesia onset time (T_onset) was defined as the time elapsing from the end of injection until lateral recumbency and recorded. Induction quality was scored (Table 2) and assessed using a VAS (from 0 corresponding to best induction to 100 mm corresponding to worst induction). Oxygen was then supplemented (10 L min^−1^) via a nasal tube (10 mm ED) introduced in the ventral meatus of the dependent nostril, up to the lateral canthus of the eye. In case of apnea lasting for more than 1 minute, endo-tracheal intubation would be performed and ventilation assisted by using a Hudson valve. The time elapsed between the end of S-ketamine administration (induction) and first surgical incision (T_incision), first (T_testis_1) and second (T_testis_2) testicle removal, end of surgery (T_surgery), as well as appearance of brisk palpebral reflex (T_palpebral), first purposeful movement (T_movement), sternal recumbency (T_sternal) and standing position (T_standing) was recorded. Lidocaine 2% (10–15 mL) was injected by the surgeon into the testicle and the spermatic cord before each testicle had been emasculated. Depth of anaesthesia was continuously evaluated and occurrence of palpebral reflex, blinking, nystagmus, sweating, tears production, twitching, ear movement and swallowing were recorded and if judged inadequate as revealed by tail, head or limb movements, or excessive muscular tension, 1 mg/kg S-ketamine was administered IV up to 2 times. Thiopental (1–2 mg/kg) was administered IV if further deepening was deemed necessary. In case of increased muscular tone during the surgical procedure without purposeful movements, 0.02 mg/kg romifidine was administered once IV. All animals were monitored clinically including recording of PR and RR, and instrumented (Microcap Plus®, Oridion) with portable capnography (upper nostril) and pulse oxymetry (tongue) until sternal recumbency was achieved. All the horses recovered without assistance, but with an operator at the head to prevent uncontrolled movements or escaping. Recovery quality was scored (Table 2) and assessed via VAS (from 0 corresponding to best recovery to 100 mm corresponding to worst recovery). Once the horses recovered a standing position, HR and RR were recorded. The scores were always attributed by the same investigator, except the score for the quality of surgical condition (Table 2) attributed by the surgeon.

**Table 2:** **Evaluation of the quality of anaesthesia induction, surgical conditions and recovery phase**

**Parameter**	**Score**	**Description**
Induction score	1 (Excellent)	Sinks smoothly to the ground, no forward-backward movement, no excitement
	2 (Good)	Sinks smoothly to the ground, slight forward-backward movement, slight head or limb twitching
	3 (Fair)	Inadequate relaxation, forward-backward movement, visible ataxia, twitching, slight limb paddling
	4 (Poor)	Inadequate relaxation, considerable movement and ataxia, marked limb paddling
	5 (Very poor)	Failure to reach recumbency
Recovery score	1 (Excellent)	Quiet, 1 or 2 coordinated efforts to sternal and standing positions, minimal to no ataxia once standing
	2 (Good)	Quiet, 1 or 2 slightly uncoordinated efforts to sternal and standing positions, mild ataxia once standing
	3 (Fair)	Multiple (>2) quiet attempts to sternal and standing positions, mild to considerable ataxia once standing
	4 (Poor)	Multiple (>2) uncoordinated attempts to sternal and standing positions
	5 (Very poor)	Recovery resulting in major injury
Surgical score	1 (Excellent)	Good muscle relaxation, easy extraction of the testicle
	2 (Good)	Moderate retraction of the cremaster muscle
	3 (Fair)	Difficult extraction of the testicle
	4 (Poor)	Movements of the horse during surgery, reinjection required

### Sampling and analytical procedures

Venous blood (10 mL) was sampled from the catheter and collected in lithium heparinized tubes before the injection of S-ketamine (−2) and at 2, 4, 8, 13 and 20 minutes after the end of S-ketamine injection. In case of a second injection of S-ketamine, further samples at 2, 4 and 8 minutes following the reinjection were collected. Owing to clinical context, slight variations of collection times were allowed and deviations were reported. After collection, the samples were placed in an isolated package on ice until centrifugation was performed. Plasma was frozen at −80°C until the assay was performed. Concentrations of S-ketamine and S-norketamine were determined using enantioselective capillary electrophoresis (CE) according to the techniques described by Theurillat and colleagues [17,18] using the extraction procedure described in Bergadano *et al*. [19] with phosphoric acid for acidification and reconstitution in 1:1 diluted running buffer without chiral selector. The pH 2.5 Tris/phosphate running buffer contained 2% (w/v) highly sulfated γ-cyclodextrin (Beckman Coulter, Fullerton, California, USA) as chiral selector. The limit of quantification (LOQ) was set at 0.5 μM (0.12 μg mL^−1^).

### Pharmacokinetic modeling

Each step of the pharmacokinetic modeling was performed with commercially available software (Phoenix™ WinNonlin® 6.2, Pharsight Inc.). Based on data fit, diagnostic plots, Akaike information criterion (AIC) and residuals analysis, the most suitable standard mammillary multi-compartmental model was determined for S-ketamine plasma concentrations of each individual. This was performed in order to obtain initial estimates for the population modeling. Non-compartmental analysis for S-ketamine and S-norketamine concentrations were also performed to orient initial estimates. Then, different combined parent-metabolite models of the population pharmacokinetic data were evaluated using different Non-Linear Mixed Effects algorithms. The model was firstly fit to the data from the initial single bolus of S-ketamine in order to determine the best applicable procedure and the parent-metabolite relationship. The model was subsequently refined by simultaneously fitting all available concentration-time profiles (including re-dosing). The final estimates were evaluated based on data fit, adequacy to initial estimates, diagnostic parameters and residual analysis. Based on the final model, a simulation for maintaining steady state concentration for S-ketamine was carried out, targeting a plasma concentration compatible with the maintenance of surgical level of anaesthesia.

### Statistical analysis

The SigmaStat 3.5 (Point Richmond, CA, USA) software package was used to perform statistical evaluation and significance was reached at *P* = 0.05. Due to the small number of sampling, all the data are presented as median and IQR independently of their distribution.

Effect of time on PR and RR was tested with one way ANOVA test in RANK for repeated measures. Spearman rank order test was used to test: 1) correlations between quality of sedation, induction and recovery (score and VAS) in all horses (n = 28) and in the horses that did not receive any additional drug during the procedure (n = 20); 2) correlation between time at which clinical signs of lightening of anaesthesia appeared and duration of anaesthesia; 3) correlation between T_movement and predicted plasma concentrations of S-ketamine at T_movement in all horses that did not move before the end of surgery; 4) correlation between T_movement and the area under the curves of the predicted concentration of S-ketamine against time from 2 to 20 minutes (AUC S-k _2–20_).

Differences of measured S-ketamine plasma concentrations at 2, 4, 8 minutes after administration between the horses receiving one or two boluses were tested by a 2 way-ANOVA test for repeated measures.

## Results

### Clinical results

Twenty-eight colts with a median age of 30 months (IQR 24–42) and a median weight of 453 kg (IQR 386.5-510.5) were anaesthetized and castrated. An adequate sedation score (<3) was reached after the first administration of romifidine in 17 horses (61%), as judged at a median time of 23 minutes (IQR 20.25-27) after injection. One additional injection of romifidine was required by 9 horses (32%) and two by 2 horses (7%). Median T_onset was 50.5 seconds (IQR 45–57). Median induction score was 1.5 (IQR 1–2, maximum 4) where 14 horses were scored 1 (excellent), 8 horses were scored 2 (good), 5 horses were scored 3 (fair) and 1 horse (Horse #13) was scored 4 (poor). Median VAS for quality of induction was 14 mm (IQR 7.5-35.5, maximum 76).

**Figure 1:**
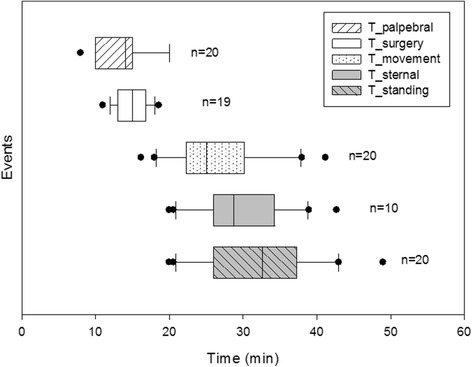
Occurrence of events after anaesthesia induction in horses that received only one bolus of S-ketamine. Time is expressed as median and 10%-25%-75%-90% interquartiles; dots represent the outsiders. Number of horses in which each event was registered is reported besides. T_palpebral: Time from anaesthesia induction until appearance of a brisk palpebral reflex; T_surgery: Time from anaesthesia induction until termination of the surgical procedure; T_movement: Time from anaesthesia induction until appearance of first movement; T_sternal: Time from anaesthesia induction until return to sternal position; T_standing: Time from anaesthesia induction until return to standing position.

For all the horses but one (horse #24), removal of the first testis (9.5 minutes, IQR 8–11.3) was possible with a single administration of S-ketamine. Twenty-one horses (75%) did not require additional S-ketamine until the end of the procedure and the T_movement was 25 minutes (IQR, 22.5-29.5). An additional bolus (1 mg/kg) was required by 7 horses at a median time of 13.5 minutes (IQR, 11.8-14.5) after movement occurred at 12.6 minutes (IQR, 11.5-14.5). Two horses (horses #17 and #24) required supplemental S-ketamine because of delayed surgery due to technical difficulties, in particular in horse #24 the extraction of the first testis was performed in 18.6 minutes. Five other horses required the second bolus during removal of the second testis. None of the horses whose exteriorization of the second testis was terminated in less than 12.5 minutes (n = 17, T_testis_2 of 12.6 minutes IQR, 11–13) required additional S-ketamine. In 2 horses, muscle relaxation was judged insufficient (horses #13 and #24) and an additional bolus of romifidine was administered intra-operatively. Horse #24 was the only horse requiring 2 additional boluses of S-ketamine and an injection of thiopental. A surgical score of 4 (poor) was attributed to the 7 horses that required additional intra-operative S-ketamine, while a score of 1 (excellent) was attributed to 17 horses, and of 2 (good) to 4 horses. Timing of the different anaesthetic events is presented in Table 3 and Figure 1. Apnea lasting longer than 60 seconds was never observed, and emergency intubation was never required.

**Table 3:** **Time (minutes) elapsed between the end of S-ketamine administration and the occurrence of significant events**

	**Group 1**		**Group 2**	
**Parameters**	**Median (IQR)**	**Range [Min-Max]**	**Median (IQR)**	**Range [Min-Max]**
T_palpebral	14 (10.5–14.7)	[8–20]	10 (9–16)	[7–20]
T_surgery	15 (13–16.5)	[11–18.6]	18.6 (15–19.8)	[14.9–23]
T_movement	25 (22.5–29.5)	[16.2–41.2]	12.6 (11.5–14.5)	[10.5–17]
T_sternal	28.8 (26–34.2)	[20–42.7]	30 (26.9–36)	[24–39.2]
T_standing	32.6 (26–37.2)	[20–49]	32.9 (30.6–39)	[27–40]

“Group 1” (n = 20) horses that received a single injection of S-ketamine.

“Group 2” (n = 7) horses that received additional injection(s) of S-ketamine during the procedure.

T_palpebral: Time from anaesthesia induction until appearance of a brisk palpebral reflex; T_surgery: Time from anaesthesia induction until termination of the surgical procedure; T_movement: Time from anaesthesia induction until appearance of first movement; T_sternal: Time from anaesthesia induction until return to sternal position; T_standing: Time from anaesthesia induction until return to standing position.

Among the clinical signs associated with recovery from general anaesthesia, only the reappearance of a strong palpebral reflex was repeatedly observed (n = 25). Among the 20 horses that during anaesthetic time did not receive any supplemental drug, a strong palpebral reflex appeared in 19 of them (95%) at a median time of 14 minutes (IQR 10.5-15) after induction of anaesthesia. Nystagmus was observed at the beginning of anaesthesia in 2 horses, when the testicles were pulled in 2 other horses, and during the whole surgical procedure in 3 horses.

Pulse-oxymetry was functioning in 8 horses only. The lowest observed SpO_2_ value was 92%. After sedation, the PR was significantly lower (*P* < 0.001) than the baseline, decreasing from 39 (IQR 36–42), to 28 (IQR 24–32) per minute. After induction, the PR returned to initial values within 10 minutes. The RR did not vary after sedation (median value of 14, IQR 13–16), but decreased significantly after induction (median value of 11, IQR 7–17.5) and progressively increased over baseline values after 18 minutes of general anaesthesia (median 20, IQR 14–28.5).

During recovery from general anaesthesia, sternal recumbency was extremely short (or absent) in 18 horses (65% of all horses). The median VAS for quality of recovery was 9.5 mm (IQR 5–24.5) with 18 horses scored as 1 (excellent), 6 horses as 2 (good), 2 horses as 3 (fair), and 1 horse as 4 (poor). None of the horses got injured during the recovery. Within the 7 horses which required additional S-ketamine, 4 horses were scored as 1 (excellent), 1 horse as 2 (good), and 1 horse as 3 (fair). Neither the induction score nor the recovery score correlated to the sedation score, but the recovery score was positively correlated to the induction score (*P* < 0.03). The same results were found analyzing the VAS.

### Pharmacokinetic results

**Figure 2:**
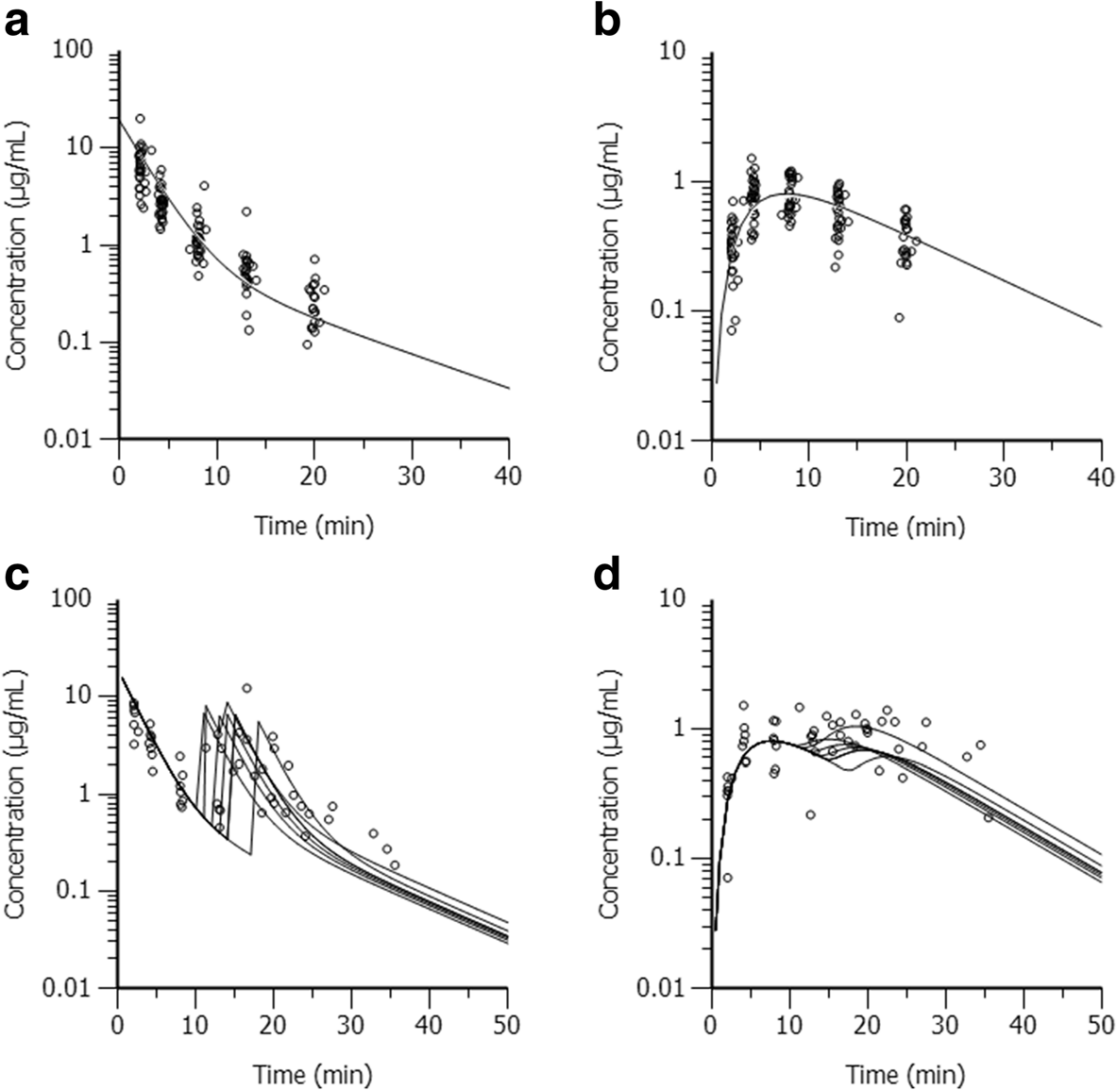
Plasma concentrations over time of S-ketamine (**a**., **c**.) and S-norketamine (**b**., **d**.). The circles represent the measured concentrations for S-ketamine (**a**., **c**.) and S-norketamine (**b**., **d**.) in horses administered S-ketamine only once (n = 21; **a**., **b**.), or twice (n = 7; **c**., **d**.). In each figure, the continuous lines represent the respective predicted concentrations for S-ketamine or S-norketamine as obtained by the final population pharmacokinetic model (a., b.: graph with mean data of 15 horses as described in Section 3.2; c., d.: individual graphs for the 7 horses).

The concentration for S-ketamine at 2 min after bolus administration (samples drawn between 2.0 and 2.6 minutes) varied between 2.61 and 20.07 μg mL^−1^ (mean ± SD: 7.01 ± 3.51 μg mL^−1^). Thereafter, the S-ketamine concentration decreased rapidly in all individuals and reached 0.48 to 2.42 μg mL^−1^ (1.15 ± 0.45 μg mL^−1^) at 8 minutes after administration (Figure 2). S-norketamine was already observed at the first sampling time; the metabolite reached peak concentration at 4 minutes (second time point, 0.88 ± 0.27 μg mL^−1^) in 14 horses and at 8 minutes (third time point, 0.86 ± 0.28 μg mL^−1^) in the other 14 individuals.

S-ketamine and S-norketamine concentration curves are graphically depicted in Figure 2. Early plasma concentrations for S-ketamine were not significantly different between horses receiving a single bolus and those requiring redosing (Figure 2).

**Figure 3:**
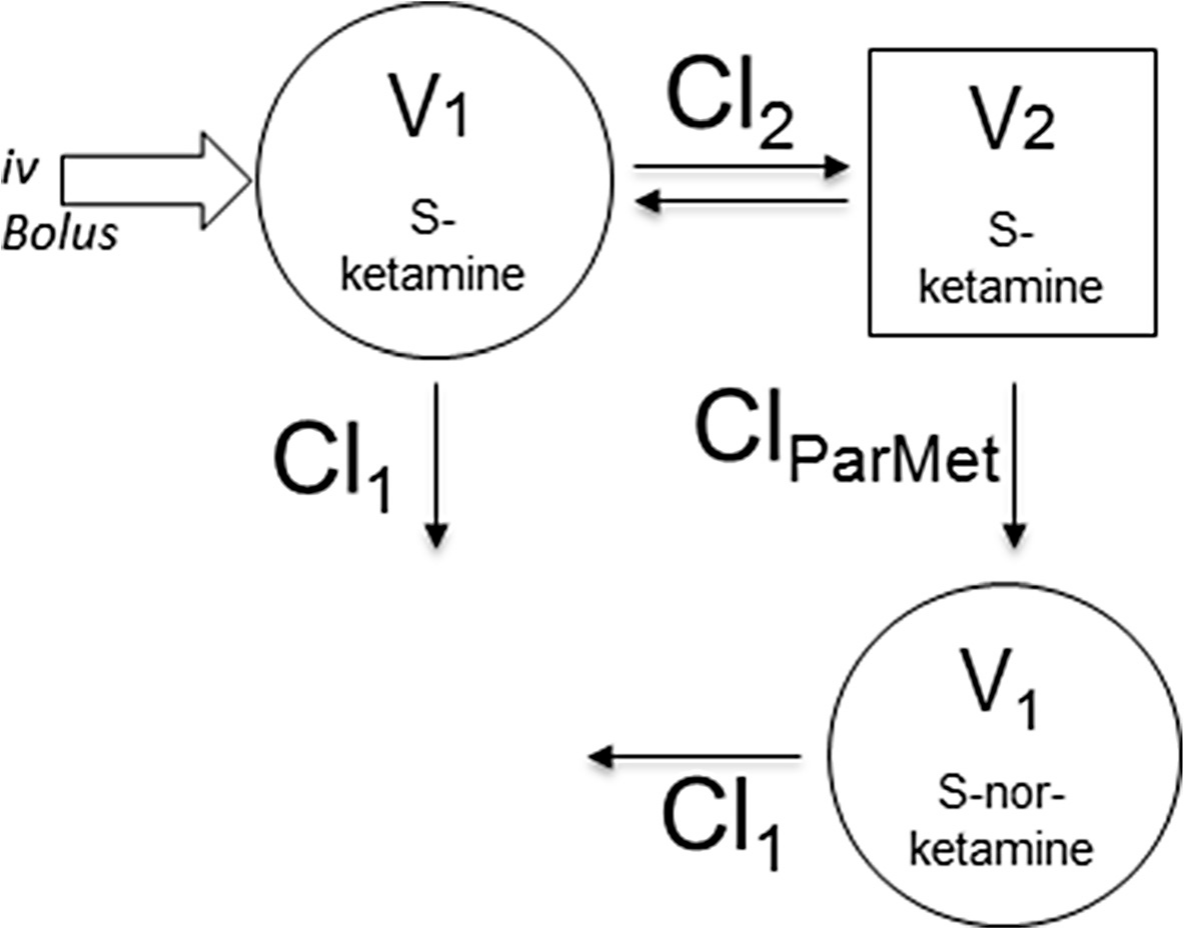
Parent-metabolite pharmacokinetic model for S-ketamine and S-norketamine. V_1_: Volume of the central compartment; Cl_1_: Clearance from the central compartment; V_2_: Volume of the peripheral compartment; Cl_2_: Clearance from the peripheral compartment; Cl_ParMet_: Clearance from S-ketamine to S-norketamine.

Due to S-ketamine re-administration in 7 horses, plasma concentrations below the LOQ in several horses, or missing samples, individual multi-compartmental modeling could be obtained only for 15 horses. For all these individuals, the time course of S-ketamine concentration was best represented by a two-compartmental model with ^1^/Ŷ as weighing factor. The one compartment model failed to capture the elimination phase of S-ketamine concentration profiles (data not shown). To describe the production of S-norketamine, the model illustrated in Figure 3 was chosen after having tested several elimination pathways and compartment arrangements. The final population parameter estimates were obtained using the iterative-2-stage method and are listed in Table 4. The fit of the prediction model to the observation data is represented in Figure 2 for horses receiving a single bolus (a.,b.) as well as for those requiring redosing (c.,d.).

**Table 4:** **Final population pharmacokinetic parameters**

**Pk paramaters**	**S-ketamine**	**S-norketamine**
V_1_ (mL/kg)	132.7	59.9
Cl_1_ (mL/kg/min)	36.3	30
V_2_ (mL/kg)	376.3	
Cl_2_ (mL/kg/min)	14.9	
Cl_ParMet_ (mL/kg/min)	21.1	
t_1/2_ (min)	8.5	8.6
Cl_tot_ (mL/kg/min)	44.5	
V_ss_ (mL/kg)	191.4	
AUC (μg/min/mL)	56.2	16.2
AUMC (μg/min^2^/mL)	241.6	272.5
MRT (min)	4.3	16.82
C_max_ (μg/mL)		0.8
T_max_ (min)		7.4

V_1_: Volume of the central compartment; Cl_1_: Clearance from the central compartment; V_2_: Volume of the peripheral compartment; Cl_2_: Clearance from the peripheral compartment; Cl_ParMet_: Clearance from S-ketamine to S-norketamine; t_1/2_: Elimination half-life; Cl_tot_: Total body clearance; V_ss_: Volume of distribution at steady-state; AUC: Area under the time course curve; AUMC: Area under the moment of the time course curve; MRT: Mean residence time; C_max_: Maximum concentration; T_max_: Time at maximum concentration.

Surgical anaesthesia tended to end between 10 and 13 minutes after bolus administration. At 8 minutes median plasma concentrations of S-ketamine were 4.64 μg mL^−1^ (IQR 3.69-5.88) and 4.14 μg mL^−1^ (IQR 3.37-6.21) in the horses receiving one and two boluses, respectively. The predicted average S-ketamine plasma concentrations were 2 μg mL^−1^ at 6 minutes and 1 μg mL^−1^ at 8.5 minutes after a single IV bolus of 2.5 mg/kg. Therefore, it was considered that maintenance of S-ketamine plasma concentration at least between 1.5 and 2 μg mL^−1^ is required for maintenance of surgical anaesthesia. Estimated elimination half-time at steady state was 2.25 minutes.

In horses not requiring the second bolus, median concentration of S-ketamine was predicted to be 0.15 (IQR 0.06-0.31) μg mL^−1^ at T_movement, and a significant negative correlation was found between S-ketamine concentration measured at T_movement and the time of movement (r_s_ -0.665 with p = .005). However the values of T_movement did not correlate with the AUC S-k_2–20._

## Discussion

As hypothesized, S-ketamine administered at 2.5 mg/kg allowed to reach surgical anaesthesia in all horses, and induction of anaesthesia was good to excellent in the large majority of the animals. However, in 5 horses (18%), surgical castration could not be completed without the administration of a second S-ketamine bolus despite the absence of surgical complications. As the total body weight was predicted using Carroll and Huntington formula, the dose of S-ketamine could have been underestimated in these five horses but their S-ketamine plasma concentrations did not differ significantly from the horses that did not require the second bolus. It is interesting to underline that in the group of horses not requiring additional S-ketamine, the noxious stimulation was terminated within 12.5 minutes, while, when the noxious stimulation was still ongoing at 12.5 minutes, the horses showed very rapidly signs of awakening and required additional S-ketamine. This observation supports that 2.5 mg/kg S-ketamine IV allowed for a mean duration of surgical anaesthesia of 12 minutes. The duration of anaesthesia observed in the present study is similar to that reported after administration of 2.2 mg/kg racemic ketamine IV in horses under experimental conditions [20] and in ponies undergoing field castration [21].

The dose of 2.5 mg/kg was selected based on a previous trial (unpublished data) indicating that 2 mg/kg appeared to be insufficient to maintain surgical anaesthesia for castration. Rossetti *et al*. [15] also reported that re-dosing was necessary at the latest 7 minutes after induction of anaesthesia when 2 mg/kg S-ketamine IV was administered under similar conditions. Such a short effect would not have allowed for performing the whole surgical procedure with a single bolus and, therefore, this dose was considered insufficient for meeting the objective of the present study. Rossetti *et al*. [15] hypothesized that occasional poor anaesthesia induction, earlier recumbency and the presence of minor excitatory effects after S-ketamine induction could have been the result of slight overdose; however, in one horse, a second dose of S-ketamine was required to obtain recumbency. In the present study, short induction times were not associated with poor induction quality, neither with excitatory effects (observed in only one horse). Therefore, an increase of induction dose to 2.5 mg/kg IV can be safely suggested when a surgical depth of anaesthesia has to be maintained for longer than 10 minutes.

Further administration of S-ketamine did not compromise either surgery or recovery quality. While the duration of a surgical depth of anaesthesia was prolonged for horses receiving a second bolus of S-ketamine (1 mg/kg), the time between induction of general anaesthesia and standing position was not significantly longer.

During the trial, pulse oximetry had high incidence of failure. Presence of sunlight, potential peripheral vasoconstriction and hypertension, recorded irregularities in the heart rhythm could explain its poor function; thus, ensuring clinical monitoring and oxygen supplementation was deemed essential in the present trial.

The pharmacokinetic profiles of ketamine and S-ketamine have been characterized in previous studies as being based on one-, two- or three compartments [22-24]. In the present investigation, as in others [25-28] the time course of S-ketamine was clearly at least bi-exponential in all individuals, and the addition of a third compartment did not improve significantly the diagnostic plots, increasing the AICs. However, in several horses the number of samples was insufficient to characterize a tri-exponential curve. The limited sampling time was due to technical reasons, namely the difficulty to perform further blood collection at fixed time points during recovering from general anaesthesia in field conditions. Moreover, the last measured plasma concentrations approximated the LOQ, therefore further samples could not have guaranteed gathering pharmacokinetic information. The small amounts of time points over a short time do not allow characterizing adequately the presence of a third compartment and the estimation of a terminal half-time. Simultaneously, a single compartment was sufficient to model the concentration profile of S-norketamine. In two previous studies applying a parent-metabolite model to characterize ketamine and its metabolites [28,29], the only possible elimination pathway given for ketamine was the production of norketamine without direct elimination from the central compartment. In the present study, the addition of a direct clearance pathway for S-ketamine from its central compartment beside conversion to S-norketamine improved the model fit for both compounds, supporting the rapid elimination of S-ketamine unchanged in urine, as already largely documented in equine and in other species for ketamine [18,30]. In the previous literature, different models of norketamine conversion were applied: Lankveld *et al*. [28] modeled the conversion of norketamine from the central compartment while Herd and colleagues [24] applied additional intermediate compartments to account for the transit time between ketamine clearance and norketamine production. In the present study, conversion to S-norketamine from the peripheral compartment appeared a simple method improving data fit and avoiding over-parameterization. Despite differences in model structures and study design, the overall pharmacokinetic parameters for volume of distribution and clearance were similar to previous studies [6,7,28,31]. However, pharmacokinetic investigations including larger populations under different settings are still required before it will be possible to generalize the pharmacokinetic profile of S-ketamine and its metabolites in horses.

Waterman and colleagues [26] proposed that after a bolus of 2.2 mg/kg racemic ketamine, horses may return to consciousness at a ketamine concentration of 1 μg mL^−1^. In the present study, levels of anaesthesia became incompatible with surgical procedure at S-ketamine concentration close to 1 μg mL^−1^ and signs of arousal (estimated around 13 minutes when no bolus was re-administered) occured close to 0.6 μg ml^−1^. However, the variability of the observed plasma concentrations of S-ketamine at T_movement and its correlation to anaesthesia duration do not support the definition of a clear cut-off value to predict the termination of the effect.

According to the pharmacokinetic simulation, in order to maintain surgical level of anaesthesia with S-ketamine administered at constant rate infusion, an infusion around 70 μg kg^−1^ min^−1^ has to be started immediately after the induction of anaesthesia. However, until further investigations will be provided, close monitoring to adapt drug delivery is mandatory, taking into account the rapid elimination of S-ketamine, the inter-individual variability and the rapid loss of effect over a narrow range of concentrations. Marntell *et al*. [32] reported that premedication and quality of sedation can influence the duration of dissociative anaesthesia for short surgical procedures. In the present study, a dosage of romifidine between 0.1 and 0.145 mg/kg was administered to target an appropriate and comparable quality of sedation for all horses. It is likely that reducing the manipulations between sedation and induction, a lower dose of romifidine could be used in some animals; however, due to the frantic temperament of most colts, an intravenous catheter could not have been placed before sedation in the majority of the animals. According to Kerr *et al*. [19], the duration of anaesthesia after a combination of ketamine and diazepam is prolonged if romifidine instead of xylazine is administered in premedication. The use of romifidine appears a reasonable suggestion for premedication when the effect of the anaesthetic drug is short, as for S-ketamine. Moreover, the long sedative effects achieved with romifidine [33] represent an advantage when the interval between sedation and induction cannot be standardized. The association of alpha 2 agonists and opioids has already demonstrated a synergistic effect in clinical and experimental models without an increase in adverse effects from the single drugs [34-38]. Thus, it seems unlikely that a different premedication (based on current drugs routinely used for pre-anaesthetic sedation in horses) would be able to further increase the duration of anaesthesia after 2.5 mg/kg IV S-ketamine.

In the present study, the only clinical sign occurring previous to the first movement in a majority of horses was the return of a brisk palpebral reflex. Arousal signs were otherwise minimal. When the surgical procedure lasted longer than 13 minutes, noxious stimulation triggered the recovery and purposeful movements occurred suddenly.

## Conclusions

The administration of 2.5 mg/kg IV S-ketamine after adequate premedication provided good quality of induction and recovery and a duration of action similar to racemic ketamine at the dose of 2.2 mg/kg. The short duration of surgical anaesthesia promoted by the single injection of S-ketamine might be insufficient to complete surgical castration. Taking into account the pharmacologic behavior of S-ketamine, namely the rapid elimination and the rapid loss of effect over a narrow range of concentrations, a sudden return of consciousness has to be then foreseen once the first 10 minutes after injection are elapsed. Therefore, if the surgical procedure is not terminated yet, need for redosing has to be expected 10 to 15 minutes after injection.

### Consent

Oral informed consent was obtained by BW from the patients ‘owners for the publication of this report and any accompanying images.
